# Poly[bis­(μ_3_-dodecyl sulfato)­calcium]

**DOI:** 10.1107/S1600536810020659

**Published:** 2010-06-05

**Authors:** Genta Sakane, Masahiro Tomohara, Yasuhiro Katayama, Koya Hayashi

**Affiliations:** a Department of Chemistry, Okayama University of Science, Ridai-cho, Okayama 700-0005, Japan

## Abstract

In the title compound [Ca(C_12_H_25_O_4_S)_2_]_*n*_, the unique Ca^II^ ion lies on an inversion center and is coordinated in a slightly distorted octa­hedral environment by six O atoms from dodecyl sulfate anions. The crystal structure is based on hydro­carbon (dodecyl sulfate) layers which sandwich the Ca^II^ ions. Within the layers, the hydro­carbon zigzag chains are parallel to one another and inter­act *via* van der Waals forces.

## Related literature

For studies of the title compound using atomic force microscopy, see: Rodriguez *et al.* (2002 ▶). For the Krafft point of the title compound, see: Hato & Shinoda (1973 ▶).
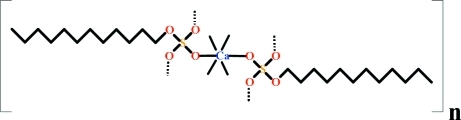


## Experimental

### 

#### Crystal data

[Ca(C_12_H_25_O_4_S)_2_]
*M* *_r_* = 570.84
Triclinic, 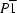

*a* = 5.3888 (3) Å
*b* = 5.3834 (3) Å
*c* = 29.1922 (16) Å
α = 93.4321 (19)°
β = 90.099 (4)°
γ = 118.393 (5)°
*V* = 743.22 (7) Å^3^
*Z* = 1
Mo *K*α radiation
μ = 0.39 mm^−1^
*T* = 93 K
0.50 × 0.10 × 0.10 mm

#### Data collection

Rigaku R-AXIS IV diffractometer
4361 measured reflections
2500 independent reflections
2396 reflections with *I* > 2σ(*I*)
*R*_int_ = 0.031

#### Refinement

*R*[*F* ^2^ > 2σ(*F* ^2^)] = 0.031
*wR*(*F* ^2^) = 0.088
*S* = 1.08
2500 reflections
235 parameters
All H-atom parameters refined
Δρ_max_ = 0.39 e Å^−3^
Δρ_min_ = −0.43 e Å^−3^

### 

Data collection: *PROCESS-AUTO* (Rigaku, 1998 ▶); cell refinement: *PROCESS-AUTO*; data reduction: *Yadokari-XG 2009* (Kabuto *et al.*, 2009 ▶); program(s) used to solve structure: *SIR2004* (Burla *et al.*, 2005 ▶); program(s) used to refine structure: *SHELXL97* (Sheldrick, 2008 ▶); molecular graphics: *Yadokari-XG 2009* and *VESTA* (Momma *et al.*, 2008 ▶); software used to prepare material for publication: *Yadokari-XG 2009*.

## Supplementary Material

Crystal structure: contains datablocks I, global. DOI: 10.1107/S1600536810020659/lh5055sup1.cif

Structure factors: contains datablocks I. DOI: 10.1107/S1600536810020659/lh5055Isup2.hkl

Additional supplementary materials:  crystallographic information; 3D view; checkCIF report

## Figures and Tables

**Fig. 1.**
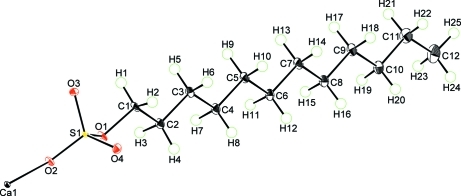
The asymmetric unit of (I) with atom labels and 50% probability displacement ellipsoids for non-H atoms. H atoms are shown as spheres of arbitary radius.

**Fig. 2.**
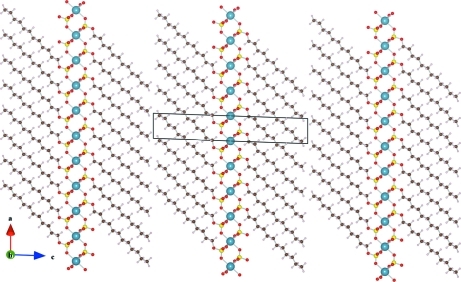
The packing of (I), viewed along the b axis, showing hydrocarbon (dodecyl sulfate) layers which sandwich Ca atoms.

## supplementary crystallographic information

### Comment

The crystal of the title compound, (I) (Fig. 1), is mechanically flexible because (I) is a two-dimensional layered compound which is characterized by strong covalent bonds and coordination bonds within the layers and weak van der Waals forces between the layers (Fig. 2).

### Experimental

The title compound was prepared by the addition of CaCl_2_ to sodium dodecyl sulfate (SDS) in a water-ethanol mixed solvent. A crystal suitable for single-crystal X-ray diffraction was selected directly from the prepared sample.

### Refinement

All H atoms were located in a difference map as peaks of density and refined with isotropic thermal parameters; the range of C—H bond lengths is 0.94 (2)-1.03 (3) Å.

### Crystal data

**Table:**

[Ca(C_12_H_25_O_4_S)_2_]	*Z* = 1
*M**_r_* = 570.84	*F*(000) = 310
Triclinic, *P*1	*D*_x_ = 1.275 Mg m^−^^3^
Hall symbol: -P 1	Mo *K*α radiation, λ = 0.71069 Å
*a* = 5.3888 (3) Å	Cell parameters from 3581 reflections
*b* = 5.3834 (3) Å	θ = 1.4–25.5°
*c* = 29.1922 (16) Å	µ = 0.39 mm^−^^1^
α = 93.4321 (19)°	*T* = 93 K
β = 90.099 (4)°	Needle, colourless
γ = 118.393 (5)°	0.50 × 0.10 × 0.10 mm
*V* = 743.22 (7) Å^3^	

### Data collection

**Table:**

Rigaku R-AXIS IV diffractometer	2396 reflections with *I* > 2σ(*I*)
Radiation source: rotating-anode X-ray	*R*_int_ = 0.031
Graphite Monochromator	θ_max_ = 25.5°, θ_min_ = 1.4°
Detector resolution: 10.0 pixels mm^-1^	*h* = −5→5
ω scans	*k* = −6→6
4361 measured reflections	*l* = −35→35
2500 independent reflections	

### Refinement

**Table:**

Refinement on *F*^2^	Primary atom site location: structure-invariant direct methods
Least-squares matrix: full	Secondary atom site location: difference Fourier map
*R*[*F*^2^ > 2σ(*F*^2^)] = 0.031	Hydrogen site location: inferred from neighbouring sites
*wR*(*F*^2^) = 0.088	All H-atom parameters refined
*S* = 1.08	*w* = 1/[σ^2^(*F*_o_^2^) + (0.0512*P*)^2^ + 0.4436*P*] where *P* = (*F*_o_^2^ + 2*F*_c_^2^)/3
2500 reflections	(Δ/σ)_max_ = 0.001
235 parameters	Δρ_max_ = 0.39 e Å^−^^3^
0 restraints	Δρ_min_ = −0.43 e Å^−^^3^

### Special details

**Table:**

Geometry. All esds (except the esd in the dihedral angle between two l.s. planes) are estimated using the full covariance matrix. The cell esds are taken into account individually in the estimation of esds in distances, angles and torsion angles; correlations between esds in cell parameters are only used when they are defined by crystal symmetry. An approximate (isotropic) treatment of cell esds is used for estimating esds involving l.s. planes.
Refinement. Refinement of *F*^2^ against ALL reflections. The weighted *R*-factor wR and goodness of fit *S* are based on *F*^2^, conventional *R*-factors *R* are based on *F*, with *F* set to zero for negative *F*^2^. The threshold expression of *F*^2^ > σ(*F*^2^) is used only for calculating *R*-factors(gt) etc. and is not relevant to the choice of reflections for refinement. *R*-factors based on *F*^2^ are statistically about twice as large as those based on *F*, and *R*- factors based on ALL data will be even larger.

### Fractional atomic coordinates and isotropic or equivalent isotropic displacement parameters (Å^2^)

**Table:**

	*x*	*y*	*z*	*U*_iso_*/*U*_eq_	
Ca1	−1.0000	−0.5000	0.5000	0.00470 (14)	
S1	−0.37678 (7)	−0.17886 (7)	0.442177 (11)	0.00452 (13)	
O1	−0.2874 (2)	−0.2127 (2)	0.39188 (3)	0.0088 (3)	
O2	−0.6786 (2)	−0.3652 (2)	0.44018 (3)	0.0082 (2)	
O3	−0.3024 (2)	0.1177 (2)	0.45120 (3)	0.0086 (2)	
O4	−0.2238 (2)	−0.2644 (2)	0.47274 (3)	0.0106 (3)	
C1	0.0057 (3)	−0.0139 (3)	0.38163 (5)	0.0099 (3)	
C2	0.0898 (3)	−0.1473 (3)	0.34179 (5)	0.0098 (3)	
C3	0.3718 (4)	0.0621 (3)	0.32332 (5)	0.0106 (3)	
C4	0.4633 (4)	−0.0705 (3)	0.28353 (5)	0.0110 (3)	
C5	0.7364 (4)	0.1365 (3)	0.26211 (5)	0.0108 (3)	
C6	0.8242 (4)	0.0010 (3)	0.22225 (5)	0.0113 (3)	
C7	1.0951 (4)	0.2073 (3)	0.20023 (5)	0.0121 (3)	
C8	1.1836 (4)	0.0689 (3)	0.16093 (5)	0.0121 (3)	
C9	1.4510 (4)	0.2760 (4)	0.13817 (5)	0.0135 (4)	
C10	1.5462 (4)	0.1376 (4)	0.09979 (5)	0.0141 (4)	
C11	1.8122 (4)	0.3468 (4)	0.07713 (6)	0.0202 (4)	
C12	1.9121 (5)	0.2066 (5)	0.03986 (6)	0.0248 (4)	
H1	−0.004 (5)	0.149 (5)	0.3738 (8)	0.030*	
H2	0.129 (5)	0.029 (5)	0.4080 (8)	0.030*	
H3	−0.066 (5)	−0.220 (5)	0.3174 (8)	0.030*	
H4	0.095 (5)	−0.318 (5)	0.3516 (8)	0.030*	
H5	0.352 (5)	0.218 (5)	0.3138 (8)	0.030*	
H6	0.512 (5)	0.139 (5)	0.3479 (8)	0.030*	
H7	0.302 (5)	−0.159 (5)	0.2590 (8)	0.030*	
H8	0.484 (5)	−0.230 (5)	0.2944 (8)	0.030*	
H9	0.708 (5)	0.296 (5)	0.2513 (7)	0.030*	
H10	0.886 (5)	0.224 (5)	0.2861 (8)	0.030*	
H11	0.670 (5)	−0.078 (5)	0.1986 (8)	0.030*	
H12	0.850 (5)	−0.157 (5)	0.2328 (8)	0.030*	
H13	1.065 (5)	0.367 (5)	0.1885 (8)	0.030*	
H14	1.251 (5)	0.302 (5)	0.2243 (8)	0.030*	
H15	1.032 (5)	−0.013 (5)	0.1384 (8)	0.030*	
H16	1.216 (5)	−0.082 (5)	0.1722 (8)	0.030*	
H17	1.415 (5)	0.426 (5)	0.1260 (8)	0.030*	
H18	1.606 (5)	0.369 (5)	0.1619 (8)	0.030*	
H19	1.399 (5)	0.041 (5)	0.0767 (8)	0.030*	
H20	1.580 (5)	−0.007 (5)	0.1123 (8)	0.030*	
H21	1.773 (5)	0.488 (5)	0.0641 (8)	0.030*	
H22	1.955 (5)	0.437 (5)	0.1011 (8)	0.030*	
H23	1.771 (6)	0.110 (5)	0.0152 (9)	0.037*	
H24	1.940 (6)	0.050 (5)	0.0518 (8)	0.037*	
H25	2.097 (6)	0.344 (5)	0.0273 (8)	0.037*	

### Atomic displacement parameters (Å^2^)

**Table:**

	*U*^11^	*U*^22^	*U*^33^	*U*^12^	*U*^13^	*U*^23^
Ca1	0.0033 (2)	0.0043 (2)	0.0060 (2)	0.00128 (17)	0.00087 (15)	0.00124 (15)
S1	0.0037 (2)	0.0044 (2)	0.00495 (19)	0.00141 (16)	0.00119 (13)	0.00066 (13)
O1	0.0050 (6)	0.0096 (6)	0.0069 (5)	−0.0003 (4)	0.0035 (4)	−0.0005 (4)
O2	0.0053 (6)	0.0081 (5)	0.0079 (5)	0.0005 (4)	0.0019 (4)	0.0005 (4)
O3	0.0090 (6)	0.0064 (5)	0.0099 (5)	0.0034 (4)	0.0004 (4)	−0.0006 (4)
O4	0.0099 (6)	0.0116 (5)	0.0120 (5)	0.0061 (5)	−0.0008 (4)	0.0036 (4)
C1	0.0061 (8)	0.0096 (8)	0.0106 (7)	0.0008 (6)	0.0044 (6)	0.0015 (6)
C2	0.0083 (8)	0.0110 (8)	0.0091 (7)	0.0039 (6)	0.0033 (6)	0.0005 (6)
C3	0.0091 (9)	0.0122 (8)	0.0099 (7)	0.0045 (6)	0.0036 (6)	0.0023 (6)
C4	0.0096 (8)	0.0130 (8)	0.0107 (7)	0.0057 (6)	0.0037 (6)	0.0015 (6)
C5	0.0094 (9)	0.0127 (8)	0.0103 (7)	0.0051 (7)	0.0043 (6)	0.0026 (6)
C6	0.0096 (9)	0.0149 (8)	0.0106 (7)	0.0066 (7)	0.0033 (6)	0.0019 (6)
C7	0.0094 (8)	0.0145 (8)	0.0116 (7)	0.0050 (7)	0.0050 (6)	0.0016 (6)
C8	0.0110 (9)	0.0150 (8)	0.0110 (7)	0.0066 (7)	0.0038 (6)	0.0019 (6)
C9	0.0117 (9)	0.0168 (9)	0.0128 (7)	0.0075 (7)	0.0064 (6)	0.0024 (6)
C10	0.0130 (9)	0.0187 (9)	0.0119 (7)	0.0087 (7)	0.0042 (6)	0.0009 (6)
C11	0.0215 (10)	0.0244 (10)	0.0181 (8)	0.0131 (8)	0.0113 (7)	0.0060 (7)
C12	0.0256 (11)	0.0392 (12)	0.0179 (9)	0.0216 (9)	0.0116 (7)	0.0067 (8)

### Geometric parameters (Å, °)

**Table:**

Ca1—O4^i^	2.2955 (11)	C4—H8	0.99 (2)
Ca1—O4^ii^	2.2956 (11)	C5—C6	1.529 (2)
Ca1—O3^iii^	2.3177 (10)	C5—H9	1.01 (2)
Ca1—O3^iv^	2.3178 (11)	C5—H10	0.98 (2)
Ca1—O2	2.3544 (10)	C6—C7	1.524 (2)
Ca1—O2^v^	2.3544 (10)	C6—H11	0.99 (2)
Ca1—S1^v^	3.4589 (4)	C6—H12	0.99 (2)
Ca1—S1	3.4589 (4)	C7—C8	1.528 (2)
S1—O4	1.4474 (11)	C7—H13	1.03 (2)
S1—O2	1.4476 (11)	C7—H14	1.00 (2)
S1—O3	1.4558 (11)	C8—C9	1.525 (2)
S1—O1	1.5732 (10)	C8—H15	0.96 (2)
O1—C1	1.4703 (18)	C8—H16	0.98 (2)
O3—Ca1^vi^	2.3177 (10)	C9—C10	1.530 (2)
O4—Ca1^vii^	2.2956 (11)	C9—H17	1.00 (2)
C1—C2	1.512 (2)	C9—H18	0.99 (2)
C1—H1	0.94 (2)	C10—C11	1.522 (2)
C1—H2	0.96 (2)	C10—H19	0.96 (2)
C2—C3	1.522 (2)	C10—H20	0.97 (2)
C2—H3	1.01 (2)	C11—C12	1.528 (2)
C2—H4	0.99 (2)	C11—H21	0.98 (2)
C3—C4	1.529 (2)	C11—H22	0.96 (2)
C3—H5	0.95 (2)	C12—H23	0.97 (3)
C3—H6	0.96 (2)	C12—H24	1.00 (3)
C4—C5	1.525 (2)	C12—H25	1.00 (3)
C4—H7	1.03 (2)		
			
O4^i^—Ca1—O4^ii^	179.997 (1)	C2—C3—H6	109.6 (14)
O4^i^—Ca1—O3^iii^	86.81 (4)	C4—C3—H6	111.4 (15)
O4^ii^—Ca1—O3^iii^	93.19 (4)	H5—C3—H6	105.4 (19)
O4^i^—Ca1—O3^iv^	93.19 (4)	C5—C4—C3	114.00 (13)
O4^ii^—Ca1—O3^iv^	86.81 (4)	C5—C4—H7	110.0 (13)
O3^iii^—Ca1—O3^iv^	179.997 (1)	C3—C4—H7	108.4 (14)
O4^i^—Ca1—O2	92.58 (4)	C5—C4—H8	109.4 (14)
O4^ii^—Ca1—O2	87.43 (4)	C3—C4—H8	109.1 (13)
O3^iii^—Ca1—O2	87.11 (4)	H7—C4—H8	105.6 (19)
O3^iv^—Ca1—O2	92.90 (4)	C4—C5—C6	113.36 (13)
O4^i^—Ca1—O2^v^	87.43 (4)	C4—C5—H9	107.5 (14)
O4^ii^—Ca1—O2^v^	92.57 (4)	C6—C5—H9	110.2 (13)
O3^iii^—Ca1—O2^v^	92.89 (4)	C4—C5—H10	109.0 (14)
O3^iv^—Ca1—O2^v^	87.10 (4)	C6—C5—H10	111.1 (15)
O2—Ca1—O2^v^	179.998 (2)	H9—C5—H10	105.3 (19)
O4^i^—Ca1—S1^v^	80.30 (3)	C7—C6—C5	113.72 (13)
O4^ii^—Ca1—S1^v^	99.70 (3)	C7—C6—H11	108.4 (13)
O3^iii^—Ca1—S1^v^	75.13 (3)	C5—C6—H11	107.9 (14)
O3^iv^—Ca1—S1^v^	104.86 (3)	C7—C6—H12	108.5 (14)
O2—Ca1—S1^v^	161.13 (3)	C5—C6—H12	110.0 (13)
O2^v^—Ca1—S1^v^	18.87 (3)	H11—C6—H12	108.1 (19)
O4^i^—Ca1—S1	99.70 (3)	C6—C7—C8	113.32 (13)
O4^ii^—Ca1—S1	80.30 (3)	C6—C7—H13	108.1 (14)
O3^iii^—Ca1—S1	104.86 (3)	C8—C7—H13	109.9 (13)
O3^iv^—Ca1—S1	75.14 (3)	C6—C7—H14	109.6 (13)
O2—Ca1—S1	18.87 (3)	C8—C7—H14	110.4 (14)
O2^v^—Ca1—S1	161.13 (3)	H13—C7—H14	105.1 (18)
S1^v^—Ca1—S1	180.0	C9—C8—C7	113.44 (13)
O4—S1—O2	113.59 (6)	C9—C8—H15	108.9 (14)
O4—S1—O3	111.92 (6)	C7—C8—H15	107.6 (15)
O2—S1—O3	112.94 (6)	C9—C8—H16	108.1 (15)
O4—S1—O1	107.44 (6)	C7—C8—H16	110.0 (13)
O2—S1—O1	103.17 (6)	H15—C8—H16	108.7 (19)
O3—S1—O1	107.04 (6)	C8—C9—C10	113.78 (14)
O4—S1—Ca1	94.37 (5)	C8—C9—H17	107.8 (14)
O2—S1—Ca1	31.74 (4)	C10—C9—H17	110.3 (13)
O3—S1—Ca1	100.49 (5)	C8—C9—H18	108.8 (13)
O1—S1—Ca1	134.58 (4)	C10—C9—H18	108.4 (14)
C1—O1—S1	115.39 (9)	H17—C9—H18	107.7 (18)
S1—O2—Ca1	129.39 (6)	C11—C10—C9	113.27 (14)
S1—O3—Ca1^vi^	138.18 (7)	C11—C10—H19	108.9 (14)
S1—O4—Ca1^vii^	160.70 (7)	C9—C10—H19	110.8 (15)
O1—C1—C2	107.53 (12)	C11—C10—H20	109.1 (15)
O1—C1—H1	103.4 (15)	C9—C10—H20	108.9 (14)
C2—C1—H1	112.3 (14)	H19—C10—H20	105.6 (19)
O1—C1—H2	111.0 (14)	C10—C11—C12	113.14 (16)
C2—C1—H2	111.6 (15)	C10—C11—H21	107.9 (15)
H1—C1—H2	111 (2)	C12—C11—H21	110.1 (13)
C1—C2—C3	111.44 (13)	C10—C11—H22	106.5 (14)
C1—C2—H3	107.7 (14)	C12—C11—H22	109.9 (15)
C3—C2—H3	111.5 (13)	H21—C11—H22	109.1 (19)
C1—C2—H4	109.4 (13)	C11—C12—H23	112.2 (16)
C3—C2—H4	111.2 (14)	C11—C12—H24	111.7 (14)
H3—C2—H4	105.3 (19)	H23—C12—H24	103 (2)
C2—C3—C4	112.23 (13)	C11—C12—H25	112.3 (14)
C2—C3—H5	107.4 (15)	H23—C12—H25	111 (2)
C4—C3—H5	110.6 (14)	H24—C12—H25	107 (2)
			
O4^i^—Ca1—S1—O4	161.30 (7)	Ca1—S1—O1—C1	176.64 (8)
O4^ii^—Ca1—S1—O4	−18.69 (7)	O4—S1—O2—Ca1	56.79 (10)
O3^iii^—Ca1—S1—O4	−109.44 (5)	O3—S1—O2—Ca1	−72.04 (9)
O3^iv^—Ca1—S1—O4	70.56 (5)	O1—S1—O2—Ca1	172.76 (7)
O2—Ca1—S1—O4	−129.73 (10)	O4^i^—Ca1—O2—S1	112.94 (8)
O2^v^—Ca1—S1—O4	50.26 (10)	O4^ii^—Ca1—O2—S1	−67.06 (8)
S1^v^—Ca1—S1—O4	−136 (16)	O3^iii^—Ca1—O2—S1	−160.39 (9)
O4^i^—Ca1—S1—O2	−68.96 (9)	O3^iv^—Ca1—O2—S1	19.61 (9)
O4^ii^—Ca1—S1—O2	111.04 (9)	O2^v^—Ca1—O2—S1	−17 (11)
O3^iii^—Ca1—S1—O2	20.30 (9)	S1^v^—Ca1—O2—S1	180.0
O3^iv^—Ca1—S1—O2	−159.71 (9)	O4—S1—O3—Ca1^vi^	1.53 (12)
O2^v^—Ca1—S1—O2	179.998 (2)	O2—S1—O3—Ca1^vi^	131.22 (9)
S1^v^—Ca1—S1—O2	−6(16)	O1—S1—O3—Ca1^vi^	−115.93 (9)
O4^i^—Ca1—S1—O3	48.05 (5)	Ca1—S1—O3—Ca1^vi^	100.63 (9)
O4^ii^—Ca1—S1—O3	−131.95 (5)	O2—S1—O4—Ca1^vii^	80.8 (2)
O3^iii^—Ca1—S1—O3	137.31 (7)	O3—S1—O4—Ca1^vii^	−149.8 (2)
O3^iv^—Ca1—S1—O3	−42.70 (7)	O1—S1—O4—Ca1^vii^	−32.6 (2)
O2—Ca1—S1—O3	117.01 (9)	Ca1—S1—O4—Ca1^vii^	107.0 (2)
O2^v^—Ca1—S1—O3	−62.99 (9)	S1—O1—C1—C2	156.54 (11)
S1^v^—Ca1—S1—O3	111 (16)	O1—C1—C2—C3	170.34 (13)
O4^i^—Ca1—S1—O1	−78.87 (7)	C1—C2—C3—C4	178.97 (14)
O4^ii^—Ca1—S1—O1	101.13 (7)	C2—C3—C4—C5	176.61 (13)
O3^iii^—Ca1—S1—O1	10.38 (7)	C3—C4—C5—C6	−179.75 (13)
O3^iv^—Ca1—S1—O1	−169.62 (7)	C4—C5—C6—C7	179.24 (13)
O2—Ca1—S1—O1	−9.91 (10)	C5—C6—C7—C8	179.09 (13)
O2^v^—Ca1—S1—O1	170.08 (10)	C6—C7—C8—C9	178.78 (14)
S1^v^—Ca1—S1—O1	−16 (16)	C7—C8—C9—C10	178.20 (14)
O4—S1—O1—C1	−68.41 (11)	C8—C9—C10—C11	179.60 (15)
O2—S1—O1—C1	171.31 (11)	C9—C10—C11—C12	178.15 (15)
O3—S1—O1—C1	51.95 (12)		

Symmetry codes: (i) *x*−1, *y*, *z*; (ii) −*x*−1, −*y*−1, −*z*+1; (iii) *x*−1, *y*−1, *z*; (iv) −*x*−1, −*y*, −*z*+1; (v) −*x*−2, −*y*−1, −*z*+1; (vi) *x*+1, *y*+1, *z*; (vii) *x*+1, *y*, *z*.

## References

[bb1] Burla, M. C., Caliandro, R., Camalli, M., Carrozzini, B., Cascarano, G. L., De Caro, L., Giacovazzo, C., Polidori, G. & Spagna, R. (2005). *J. Appl. Cryst.* **38**, 381–388.

[bb2] Hato, M. & Shinoda, K. (1973). *Bull. Chem. Soc. Jpn*, **46**, 3889–3890.

[bb3] Kabuto, C., Akine, S., Nemoto, T. & Kwon, E. (2009). *J. Cryst. Soc. Jpn*, **51**, 218–224.

[bb4] Momma, K. & Izumi, F. (2008). *J. Appl. Cryst.* **41**, 653–658.

[bb5] Rigaku (1998). *PROCESS-AUTO* Rigaku Corporation, Tokyo, Japan.

[bb6] Rodriguez, C. H., Yuan, W.-L., Scamehorn, J. F. & O’Rear, E. A. (2002). *J. Surfact. Deterg.* **5**, 269–280.

[bb7] Sheldrick, G. M. (2008). *Acta Cryst.* A**64**, 112–122. 10.1107/S0108767307043930 18156677

